# Scrutinizing the potential role of non-coding RNAs as biomarkers in Egyptian females with early and metastatic breast cancer

**DOI:** 10.1186/s12885-025-15483-0

**Published:** 2026-01-14

**Authors:** Eman ElAlfy, Nourhan E Gaber, Madiha H Helmy, Maher A Kamel, Sara A Shaker

**Affiliations:** 1 Cancer Management and Research department, Medical Research Institute, Alexandria University, Alexandria, Egypt; 2 Department of Biochemistry, Medical Research Institute, Alexandria University, 165 El-Horeya Rd, Al Ibrahimeyah Qebli WA Al Hadrah Bahri, Qesm Bab Sharqi, Alexandria, 21561 Egypt

**Keywords:** Breast cancer, Circulating RNA, Non-Coding RNAs (miRNAs, LncRNAs), PVT1, HOTAIR, Mir-331, Mir195

## Abstract

Breast cancer (BC) is the second most frequently diagnosed malignancy worldwide and the most common cancer among Egyptian women, accounting for 38.8% of female malignancies. Early diagnosis is critical for improving outcomes; however, conventional serum tumor markers such as CEA and CA15-3 show limited sensitivity and specificity in early-stage BC. Liquid biopsy has emerged as a promising non-invasive approach, enabling detection of circulating tumor-derived products, including non-coding RNAs (ncRNAs). This study aimed to evaluate the differential expression of circulating ncRNAs in the serum of female patients with early and metastatic BC prior to initiation of chemotherapy, and to assess their associations with clinicopathological characteristics and prognostic parameters. We also sought to determine their potential utility as prognostic biomarkers for early detection of metastasis. Method: A total of fifty female participants were enrolled following ethical approval and categorized into three groups: healthy controls (*n* = 10), early-stage breast cancer patients (*n* = 21), and metastatic breast cancer patients (*n* = 19). All breast cancer cases were diagnosed with invasive disease and exhibited mixed hormone receptor status. Result: the results demonstrated that the studied ncRNAs (miRNAs and lncRNAs) effectively distinguished early BC patients from healthy controls, whereas CEA and CA15-3 did not. Conclusion: This pilot study provides preliminary evidence that PVT1, HOTAIR, miR-331, and miR-195 may represent potential diagnostic and prognostic biomarkers in BC. However, due to the limited sample size and exploratory nature of the analyses, validation in larger, independent, and prospectively collected cohorts is essential before clinical application can be established.

## Introduction

Breast cancer (BC) is the most prevalent cancer among women and the second most common malignancy worldwide, accounting for 25.2% of female cancers and approximately 0.5 million cancer-related deaths annually [1, 2]. In Egypt, BC represents the most frequent malignancy in women, with over 22,700 cases reported in 2020 and projections exceeding 46,000 by 2050 [3–5]. Despite advances in treatment, approximately 30% of patients diagnosed at early stages eventually develop distant metastases, which remain a major cause of mortality [6, 7].

The BC is a heterogeneous disease influenced by both hereditary and non-hereditary factors. Early-stage BC is confined to the breast and regional lymph nodes, whereas metastatic BC can spread to distant organs, including the lungs, liver, brain, and bones [8–10]. Clinical and pathological staging relies on tumor size (T), regional lymph node involvement (N), and distant metastasis (M), which are integrated into stage groups I–IV [11, 12]. Molecular subtyping based on hormone receptor and HER2 expression further classifies BC into luminal A, luminal B, HER2-enriched, and triple-negative subtypes [13–15].

Conventional imaging methods, such as mammography, ultrasound, and MRI, are widely used for BC detection but often identify tumors at later stages and may lack sensitivity in dense breast tissue [16–18]. Serum tumor markers, including carcinoembryonic antigen (CEA) and cancer antigen 15 − 3 (CA15-3), are limited in sensitivity and specificity for early detection, though they are useful in monitoring metastatic disease [19, 20].

Liquid biopsy has emerged as a promising non-invasive approach, enabling the detection of circulating tumor-derived products, including cell-free RNAs, which reflect tumor genetics and treatment response [21]. Among these, non-coding RNAs (ncRNAs)—classified into microRNAs (miRNAs, ~ 22 nucleotides) and long non-coding RNAs (lncRNAs, > 200 nucleotides)—play critical roles in gene regulation and cancer biology [22].

Dysregulated lncRNAs contribute to tumor progression and metastasis. For example, plasmacytoma variant translocation 1 (PVT1) acts as an oncogene in multiple cancers, while HOX antisense intergenic RNA (HOTAIR) promotes BC cell malignancy [23–25]. Circulating miRNAs are also emerging as promising diagnostic and prognostic biomarkers. miR-331 is upregulated in BC and promotes proliferation, migration, and invasion, whereas miR-195 functions as a tumor suppressor in various cancers [26–28]. This study aimed to evaluate the differential expression of Non-Coding RNAs (miRNAs, lncRNAs) in the blood of female patients with early and metastatic breast cancer before the initiation of the first cycle of chemotherapy. Also, to identify associations of the Non-Coding RNAs expression levels with the clinicopathological and prognostic parameters and validate its ability to be used as prognostic biomarkers for the early detection of metastasis that may contribute to the optimization of breast cancer management.

## Subjects and methods

### Subjects

After approval of the Ethical Committee of the Medical Research Institute, Alexandria University fifty females subjects were included in this study. Subjects included in the study were divided into three groups; Group I (healthy control females) included 10 healthy controls females, Group II (early breast cancer patients) included 21 early breast cancer patients at any age diagnosed as invasive breast cancer with mixed hormone status, and Group III (metastatic breast cancer patients) included 19 metastatic breast cancer patients at any age diagnosed as metastatic invasive breast cancer with mixed hormone status. All patients were recruited from the cancer management and research Department, Medical Research Institute, Alexandria University, Egypt.

Written informed consent was obtained from all participants before enrollment and the study was approved by the Ethical Committee of the Medical Research Institute, Alexandria University (IRB 00010526).

The inclusion criteria of the Breast cancer patients include: newly diagnosed cases female patients with pathologically confirmed breast cancer prior to receiving any chemotherapy, radiotherapy, hormonal, or targeted therapy, classified as either early-stage (non-metastatic) or metastatic according to clinical, imaging, and pathological evaluation, and with age ≥ 18 years. The patients should not have previous history of malignancy, prior chemotherapy, radiotherapy, hormonal therapy, or targeted therapy, pregnant or lactating women, known autoimmune, inflammatory, or chronic systemic diseases, or have active infection at the time of sampling.

The control subjects were age matched healthy female volunteers with no prior history of breast cancer or any other malignancy, with no current breast symptoms or abnormal breast examination and have normal breast imaging (mammography and/or ultrasound).

Data were prospectively collected from patient records, including demographics, menstrual and parity status, family and medical history, clinical findings, imaging, and pathology. Clinical examination assessed breast and lymph node status. All patients underwent bilateral mammography and breast/axillary ultrasound and imaging for metastatic workup. Pathological evaluation included FNAC and/or CNB for tumor type, grade (via Nottingham system), and biomarker status (ER, PR, HER2) using IHC, allowing molecular subtyping. Clinical staging was based on the 7th edition AJCC TNM classification.

#### TNM staging

Clinical and pathological staging was determined according to the 7th edition AJCC TNM classification, which describes: T category: primary tumor size, N category: regional lymph node involvement, and M category: presence of distant metastasis. The TNM categories were used to assign tumors to stages I, II, III, or IV, providing a standardized assessment of disease severity at diagnosis.

The blood samples were collected from patients before the initiation of the first cycle of the appropriate line of chemotherapy during the period from November 2021 to July 2022. And, Full data was recorded from patients with early and metastatic breast cancer.

## Methods

### Sample collection

Approximately 5 mL of venous blood was collected from each participant into serum gel separator tubes. Samples were centrifuged to remove cellular debris (7,000 rpm for 10–12 min), and the resulting serum was aliquoted and stored at − 80 °C until further analysis. Serum samples were used for the measurement of tumor markers (CEA and CA15-3). Additionally, serum was used for the extraction of non-coding RNAs to quantify the expression levels of PVT1, HOTAIR, miR-331, and miR-195.

### Laboratory testing

CEA was measured on Atellica^®^ IM fully automated Analyzer using a 2-site sandwich immunoassay using direct chemiluminometric technology which uses constant amounts of 2 antibodies [29]. And, CA 15 − 3 was measured on Atellica^®^ IM fully automated Analyzer using 2-step sandwich immunoassay using direct chemiluminescent technology [30].

### Relative quantification of serum level of NcRNA using quantitative real time-polymerase chain reaction (qRT-PCR)

Total RNA was isolated from 200 µL of serum using the miRNeasy Mini Kit (Qiagen, Germany, Cat. No. 217004) according to the manufacturer’s protocol. RNA was eluted in 30 µL RNase-free water and stored at − 80 °C until use. RNA concentration and purity were assessed using a NanoDrop spectrophotometer. Reverse transcription was performed using the TOPscript™ RT DryMIX (dT18/dN6 plus) kit (Enzynomics, Korea; Cat. No. RT220). For each sample, 1 µg of total RNA was used as input in a 20 µL RT reaction. The incubation conditions were as follows: 25 °C for 10 min (primer annealing). 50 °C for 30 min (cDNA synthesis). 95 °C for 5 min (enzyme inactivation). The synthesized cDNA was stored at − 20 °C for downstream applications. The qPCR was performed to quantify serum levels of lncRNA PVT1(accession no.NR_003367.3, F: TGAGAACTGTCCTTACGTGACC, R: AGAGCACCAAGACTGGCTCT), lncRNA HOTAIR (accession no.NR_047517.1 F: GGAAAGATCCAAATGGGACCA, R:CTAGGAATCAGCACGAAGCAAA), miR-331 (Cat. No.: 339325), and miR-195 (Catalog Number: MS00005933) and the miScript SYBR Green PCR Kit, which includes the miScript Universal Primer (reverse primer) and QuantiTect SYBR Green Master Mix. Each qPCR reaction was carried out in a total volume of 20 µL, containing: 10 µL SYBR Green Master Mix, 2 µL cDNA template, 1 µL forward primer, 1 µL Universal reverse primer, 6 µL nuclease-free water. All reactions were performed in duplicates using the StepOnePlus™ Real-Time PCR system (Applied Biosystems). Cycling Conditions: Initial denaturation: 95 °C for 15 min, 40 cycles of: Denaturation: 94 °C for 15 s. Annealing: 55–60 °C for 30 s (primer-specific). Extension: 72 °C for 30 s. Normalization and Data Analysis: miR-16-5p (cat no. 339306) was used as the reference gene for miRNAs, and 18 S rRNA (accession no.NR_046237.2, F: GTAACCCGTTGAACCCCATT, R: CAAGCTTATGACCCGCACTT) as the internal control for lncRNAs. Relative expression levels were calculated using the ΔΔCt (Livak) method, where: ΔCt = CT _target_ − CT _reference_, ΔΔCt = ΔCt_sample − ΔCt_control, Fold change = 2^−ΔΔCt.

### Statistical analysis of the data

For the primary comparison between early-stage and metastatic breast cancer, effect sizes were calculated using rank-biserial correlation and Cliff’s delta for nonparametric tests. ROC AUCs were interpreted as discrimination effect sizes. No post-hoc power analysis was performed; instead, effect sizes and confidence intervals were used to guide interpretation and future study planning.

Data were described and analyzed using SPSS software package version 20.0 (SPSS, Chicago, IL, USA) [31]. The data were described as median (minimum-maximum) and percentage. Quantitative data were analyzed using the Mann Whitney test and Kruskal Wallis test and the qualitative data was analyzed using Chi-squared test. Receiver operating characteristic (ROC) curve was constructed, with area under the curve (AUC) was calculated to determine the applicability of the ncRNA levels as biomarker in differentiate early from metastatic BC. Correlation studies were done using Spearman’s rank. And finally Univariate and multivariate logistic regression analysis were used to predict Metastatic breast cancer. The P value < 0.05 was reckoned to be a significant difference. Figures were prepared using GraphPad Prism 5.0 (GraphPad Software, USA).

## Results

### Demographic and clinical features of breast cancer patients and healthy controls

#### The demographic parameters of the healthy controls subjects, and early and the metastatic breast cancer patients

The demographic and clinical characteristics of participants are summarized in Table 1. Overall, the three groups were comparable in age, gravidity, parity, abortion history, comorbidities, diabetes mellitus, hypertension, family history, and menopausal status, except for other diseases, which differed significantly among the groups (*p* = 0.008).”

**Table 1:** Demographic profile of the healthy controls and BC patients

Characteristic	Healthy Controls (n=10)		Early (n=21)		Metastatic (n=19)		Test of significance (p)
	No.	%	No.	%	No.	%	
Age (Years)							H= 0.436, p= 0.804
Min. – Max.	36–63		32–64		35–69		
Mean ± SD	51.1 ± 9.62		48.62 ± 9.12		50 ± 9.95		
Median (IQR)	52.5 (42.0–60.0)		51 (42.0–55.0)		51 (43.0–53.0)		
< 50 Years	5	50	10	47.6	9	47.4	χ^2^= 0.02, p= 0.99
≤ 50 Years	5	50	11	52.4	10	52.6	
Gravidity							H= 2.656, p= 0.265
Min. – Max.	2–6		0–6		0–6		
Mean ± SD	3.7 ± 1.33		3 ± 1.87		2.42 ± 1.98		
Median (IQR)	3.5 (3.0–5.0)		3 (2.5–4.0)		3 (0.0–4.0)		
0	0	0	4	19	6	31.6	χ^2^= 4.790, p= 0.31
2	2	20	2	9.5	3	15.8	
>2	8	80	15	71.5	10	52.6	
Parity							H= 4.046, p= 0.132
Min. – Max.	2–5		0–5		0–4		
Mean ± SD	2.9 ± 0.87		2.62 ± 1.65		1.84 ± 1.46		
Median (IQR)	3 (2.0–3.0)		3 (2.0–4.0)		2 (0.0–3.0)		
Nullipara (0)	0	0.0	4	19.0	6	31.6	^MC^p= 0.129
Primipara (1)	0	0.0	0	0.0	0	0.0	
Multipara (>1)	10	100	17	81.0	13	68.4	
Abortion							^MC^p= 0.286
No previous abortion	5	50	15	71.4	13	68.4	
Once	2	20.0	4	19.0	3	15.8	
Twice	3	30.0	2	9.5	1	5.3	
Thrice	0	0	0	0	2	10.5	
Comorbidity							χ^2^= 0.817, p= 0.665
Yes	4	40	8	38.1	5	26.3	
No	6	60	13	61.9	14	73.7	
Diabetes Mellitus							^MC^p= 0.44
Yes	2	20.0	7	33.3	3	15.8	
No	8	80.0	14	66.7	16	84.2	
Hypertension							^MC^p= 0.195
Yes	4	40	6	28.6	2	10.5	
No	6	60	15	71.4	17	89.5	
Other Disease							χ^2^= 9.543, p= 0.008*
Yes	7	70.0	4	19.0	4	21.1	
No	3	30.0	17	81.0	15	78.9	
Family History							χ^2^= 4.704, p= 0.095
Positive	4	40.0	10	47.6	3	15.8	
Negative	6	60.0	11	52.4	16	84.2	
Menopausal status							χ^2^= 0.863 p= 0.649
Pre Menopause	3	30.0	10	47.6	8	42.1	
Post Menopause	7	70.0	11	52.4	11	57.9	

Data are presented as Median (IQR.) or number and percent. Clinical data were analyzed by Kruskal Wallis test, Monte Carlo exact probability, Fisher Exact and Chi-Square test

*Indicates Statistical significance at *p* ≤ 0.05

#### The clinicopathological parameters of the early and the metastatic breast cancer patients

A statistically significant difference was observed between early and metastatic breast cancer patients in the tumor grade at primary diagnosis (*P* = 0.039), cancer stage at primary diagnosis and stage at sampling (*P* = 0.001), (*P* < 0.001) respectively, and distant metastasis at baseline (M) (*P* < 0.001). While no significant difference in other clinopathological features as shown in (Table 2 and 3).

**Table 2:** Baseline (At Diagnosis) clinicopathological parameters of the breast cancer patients

Clinicopathological details	Early (n=21)		Metastatic (n=19)		Test of significance (p)
	No.	%	No.	%	
Histologic type					^FE^p= 1.0
IDC	19	90.5	18	94.7	
ILC	2	9.5	1	5.3	
Grade					^MC^p= 0.039*
X	3	14.3	0	0	
II	12	57.1	17	89.5	
III	6	28.6	2	10.5	
Tumor Size (cm)					
Mean ± SD	3.52 ± 2.48		4.07 ± 2.80		
Median (IQR)	3.25 (2.0–4.0)		3.5 (2.30–5.25)		
Tumor Size (cm)					^MC^p= 0.167
0 & ≤ 2	7	33.3	4	21.1	
> 2 & ≤ 5 cm	13	61.9	10	52.6	
> 5 cm	1	4.8	5	26.3	
Lymph node status					χ^2^= 1.200,
Positive	12	57.1	14	73.7	p= 0.273
Negative	9	42.9	5	26.3	
Lympho Vascular Invasion					χ^2^= 0.736,
Positive	14	66.7	15	78.9	p= 0.0.391
Negative	7	33.3	4	21.1	
T stage					χ^2^= 1.668,
T0,T1,T2	18	85.7	13	68.5	p= 0.197
T3,T4	3	14.3	6	31.6	
N stage					χ^2^= 5.657,
N0	9	42.9	5	26.3	p= 0.059
N1,N2	10	47.6	6	31.6	
N3	2	9.5	8	42.1	
M Stage					^FE^p<0.001*
M0	21	100	10	52.6	
M1	0	0	9	47.4	
ER status					χ^2^= 0.024,
Positive (+, ++, +++)	17	80.9	15	78.9	p= 0.876
Negative	4	19.0	4	21.1	
PR status					
Positive (+, ++, +++)	16	76.2	15	78.9	χ^2^= 0.042, p= 0.837
Negative	5	23.8	4	21.1	
HER2 status					χ^2^= 0.293,
Positive (+++)	6	28.6	4	21.1	p= 0.588
Negative	15	71.4	15	78.9	
Biological subtye					^MC^p= 0.403
Luminal A	9	42.9	10	52.6	
Luminal B-Her 2 Negative	4	19.0	1	5.3	
Luminal B-Her 2 Positive	4	19.0	4	21.1	
Her2-enriched	2	9.5	0	0	
TNBC	2	9.5	4	21.1	
Surgery					χ^2^= 0.007,
Yes	13	61.9	12	63.2	p= 0.935
No	8	38.1	7	36.8	
Type of Surgery					χ^2^= 2.172,
MRM	6	28.6	9	47.4	p= 0.338
BCS	7	33.3	3	15.8	
No Surgery	8	38.1	7	36.8	
Axillary LNs Surgery					^MC^p= 0.245
AXLND	7	33.3	2	10.5	
SLN	2	9.5	2	10.5	
No Axillary LNs Surgery	12	57.1	15	78.9	
Stage					^MC^p= 0.001*
Stage I, II	12	57.2	4	21.1	
Stage III	9	42.9	6	31.6	
Stage IV	0	0.0	9	47.4	
Diagnosis					χ^2^= 0.412,
Left Breast Cancer	10	47.6	11	57.9	p= 0.521
Right Breast Cancer	11	52.4	8	42.1	

Data are presented as Median (IQR.) or number and percent. Clinical data were analyzed by Kruskal Wallis test, *MC* Monte Carlo exact probability, *FE* Fisher Exact and Chi-Square test

Tumors (were graded according to NCCN guidelines; T size of the tumor, T0 (No Tumor), T1(≤ 2 cm), T2(2–5 cm), T3(> 5 cm), T4(infiltration of the chest wall/skin), N regional lymph nodes involvement; N1 cancer has spread to 1–3 axillary lymph node(s), N2 cancer has spread to 4–9 axillary lymph nodes, N3 cancer has spread to 10 or more axillary lymph nodes, M distant metastasis; M0 (no metastases), M1 (metastases in other organ). ER and PR, estrogen receptor and and progesterone receptor. MRM (modified radical mastectomy), BCS (Breast-conserving surgery), AXLND (Axillary Lymph nodes Surgery), SLN (sentinel lymph node)

* Indicates Statistical significance at *p* ≤ 0.05

#### Validation details for metastatic group

Metastatic group include Recurrent patients (metastatic at follow up) and De Novo patients (metastatic from the start) (Table 4).

**Table 3:** Clinicopathological parameters of the breast cancer patients at time of sampling

Clinicopathological details	Early (n=21)		Metastatic (n=19)		Test of significance (p)
	No.	%	No.	%	
ER status					χ^2^= 1.545,
Positive (+, ++, +++)	17	80.9	12	63.2	p= 0.214
Negative	4	19.0	7	36.8	
PR status					χ^2^= 1.484,
Positive (+, ++, +++)	16	76.2	11	57.9	p= 0.223
Negative	5	23.8	8	42.1	
HER2 status					χ^2^= 0.301,
Positive (+++)	6	28.6	4	21.1	p= 0.583
Negative	15	71.4	15	78.9	
Biological Classification					^MC^p= 0.423
Luminal A	9	42.9	8	42.1	
Luminal B-Her2 Negative	4	19.0	1	5.3	
Luminal B-Her2 Positive	4	19.0	3	15.8	
Her2-enriched	2	9.5	1	5.3	
TNBC	2	9.5	6	31.6	
Stage					^MC^p< 0.001*
Stage I, II	12	57.2	0	0	
Stage III	9	42.9	0	0	
Stage IV	0	0.0	19	100	

Data are presented as number and percent. Clinical data were analyzed by Kruskal Wallis test, Monte Carlo exact probability (MC), Fisher Exact (FE) and Chi-Square test

*Indicates Statistical significance at *p* ≤ 0.05. ER and PR, estrogen receptor and progesterone receptor

**Table 4:** Validation details for metastatic group

Clinopathological details	Metastatic group (n=19)	
	Number	Percent
Disease Status at the time of Sample Taken		
Recurrent (metastatic at follow up)	11	57.9
De Novo (metastatic at presentation)	8	42.1
Chemotherapy Received		
Initial Adjuvant (Reccurent)	10	52.6
Initial Neoadjuvant (Reccurent)	1	5.3
Metastatic 1^st^ Line Chemotherapy	8	42.1
(Denovo)		
Type of First Line Of Chemotherapy		
AC + TX	7	36.8
FAC	6	31.6
CMF	1	5.3
Not Received	5	26.3
Radiotherapy		
Received	7	36.8
Not Received	12	63.2
Endocrine Therapy		
Received	12	63.2
Not Received	7	36.8
Type of Metastatic		
Visceral & Non Visceral	11	57.9
Non Visceral	8	42.1
Number Of Metastatic Sites		
Single	7	36.8
Multiple	12	63.2
Metastatic Location Lung		
Yes	8	42.1
No	11	57.9
Metastatic Location Liver		
Yes	7	36.8
No	12	63.2
Metastatic Location Bone		
Yes	9	47.4
No	10	52.6
Metastatic Location LNS		
Yes	13	68.4
No	6	31.6
Other Metastatic Location		
Yes	7	36.8
No	12	63.2
PFS 1 (Years)		
Median (Min. – Max.)	2.4170 (0.00 - 19.25)	- - -
Response to therapy		
Progression	15	78.9
No progression	4	21.1

Adjuvant chemotherapy (treatment after surgery), Neoadjuvant chemotherapy (treatment before Surgery) Types of chemotherapy; *AC* Combination of Adriamycin and cyclophosphamide, *TX* Taxol, *FAC* Fluorthisacil, Adriamycin, and Cytoxan, *CMF* Cyclophosphamide, methotrexate, and fluorthisacil. Visceral metastasis (metastasis to organ inside the thorax or the abdominal cavity). *PFS* progression-free survival

### Serum expression level of tumor marker, LncRNAs and micro RNAs and ROC curve analysis

#### Tumor markers levels

There is a significant elevation in CEA and CA15.3 level in metastatic breast cancer group compared to both the healthy control group and early breast cancer group. But, there is no significant difference between early breast cancer and healthy control (Table 5).

**Table 5:** Statistical analysis of tumor markers in different studied groups

	Control (*n* = 10)	Early (*n* = 21)	Metastatic (*n* = 19)	Test of significance (*p*)
CEA level (ng/ml):				H = 17.623, *p* < 0.001*
Mean ± SD	0.78 ± 0.79	3.62 ± 10.56	21.22 ± 30.05	
Median (IQR)	0.5 (0.18–1.28)	1.19 (0.74–1.85)	10.0 (2.73–22.52)	
Significance between gps	p1 = 0.139, p2 < 0.001*, p3 = 0.002*			
CA 15.3 level (U/ml):				H = 11.892, *p* = 0.003*
Mean ± SD	14.53 ± 5.11	31.35 ± 46.11	88.51 ± 99.26	
Median (IQR)	13.65 (11.0–18.70)	19.92 (14.60–25.76)	54.1 (19.15–109.2)	
Significance between gps.	p1 = 0.095, p2 = 0.001*, p3 = 0.034*			

*H* Kruskal Wallis test, Pairwise comparison was done, *P* Significance between groups, *p1* Significance between control and early, *p2* Significance between control and metastatic, *p3* Significance between early and metastatic, *SD* Standard deviation

*Significance (*p* < 0.05)

A binary logistic regression analysis was performed to know capability of CEA tumor marker in distinguishing early from metastatic breast cancer patients by using the area under the curve (AUC) produced from receiver operator characteristic (ROC) curves to determine the sensitivity and specificity of CEA. Using cutoff value ˃2.46, the ROC curve analysis showed that CEA level had high performance with AUC of 0.807 [sensitivity of 78.95% and specificity of 85.71% (*p* < 0.001; 95% CI 0.652–0.914). By using Cutoff value ˃41.62, the ROC curve analysis of CA 15.3 showed high performance AUC of 0.716 (0.551–0.847), (*p* = 0.011), as sensitivity (95%CI) and specificity (95%CI) [57.89 (33.5–79.7) and 90.48 (69.6–98.8) respectively] (Table 6).

**Table 6:** ROC curve analysis of CEA and CA15.5 differentiating between early and metastatic stages

Marker	AUC (95% CI)	Cutoff point	Sensitivity % (95% CI)	Specificity % (95% CI)	+PV % (95% CI)	-PV % (95% CI)	*p*
CEA	0.807 (0.652–0.914)	> 2.46	78.95 (54.4–93.9)	85.71 (63.7–97.0)	83.3 (63.1–93.6)	81.8 (64.9–91.6)	< 0.001*
CA 15.5	0.716 (0.551–0.847)	> 41.62	57.89 (33.5–79.7)	90.48 (69.6–98.8)	84.6 (58.2–95.6)	70.4 (57.9–80.4)	0.011*

*AUC* Area under the curve, +*PV* Positive predictive value, -*PV* Negative predictive value

*Significant (*p* < 0.05)

#### Long noncoding RNAs levels

**Fig. 1:**
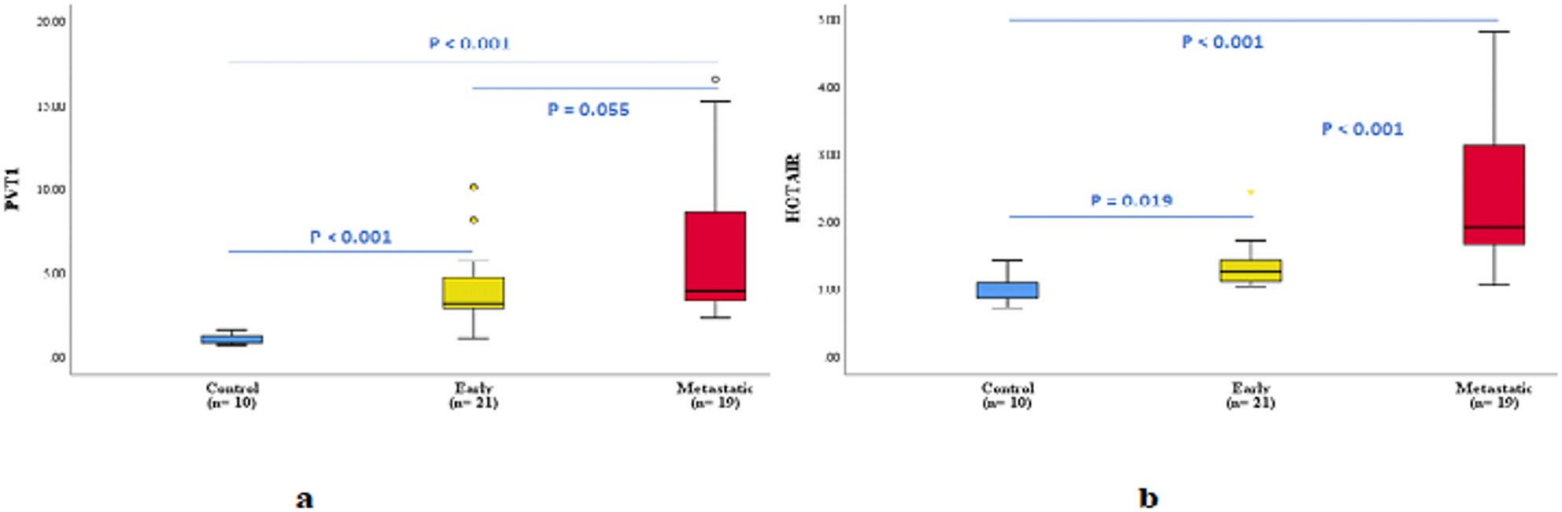
Differential circulating level of long non-coding RNAs PVT1 and HOTAIR (**a**) PVT1 circulating levels; **b** HOTAIR circulating levels

Early and metastatic breast cancer patients have significant higher circulatory levels of PVT1 compared to healthy controls, corresponding to an average fold-change of 3.8, and 6.4 respectively. Metastatic group showed non-significant higher PVT1 level compared with early group (Fig. 1a).

Investigating HOTAIR, its serum levels showed significant elevation in metastatic breast cancer patients and early breast cancer patients compared to healthy control group, with an average fold-change of 2.3, 1.3 respectively. In addition, this results showed significant higher levels of HOTAIR in metastatic patients compared with the early breast cancer patients (Fig. 1b).

#### Micro-RNAs levels

**Fig. 2:**
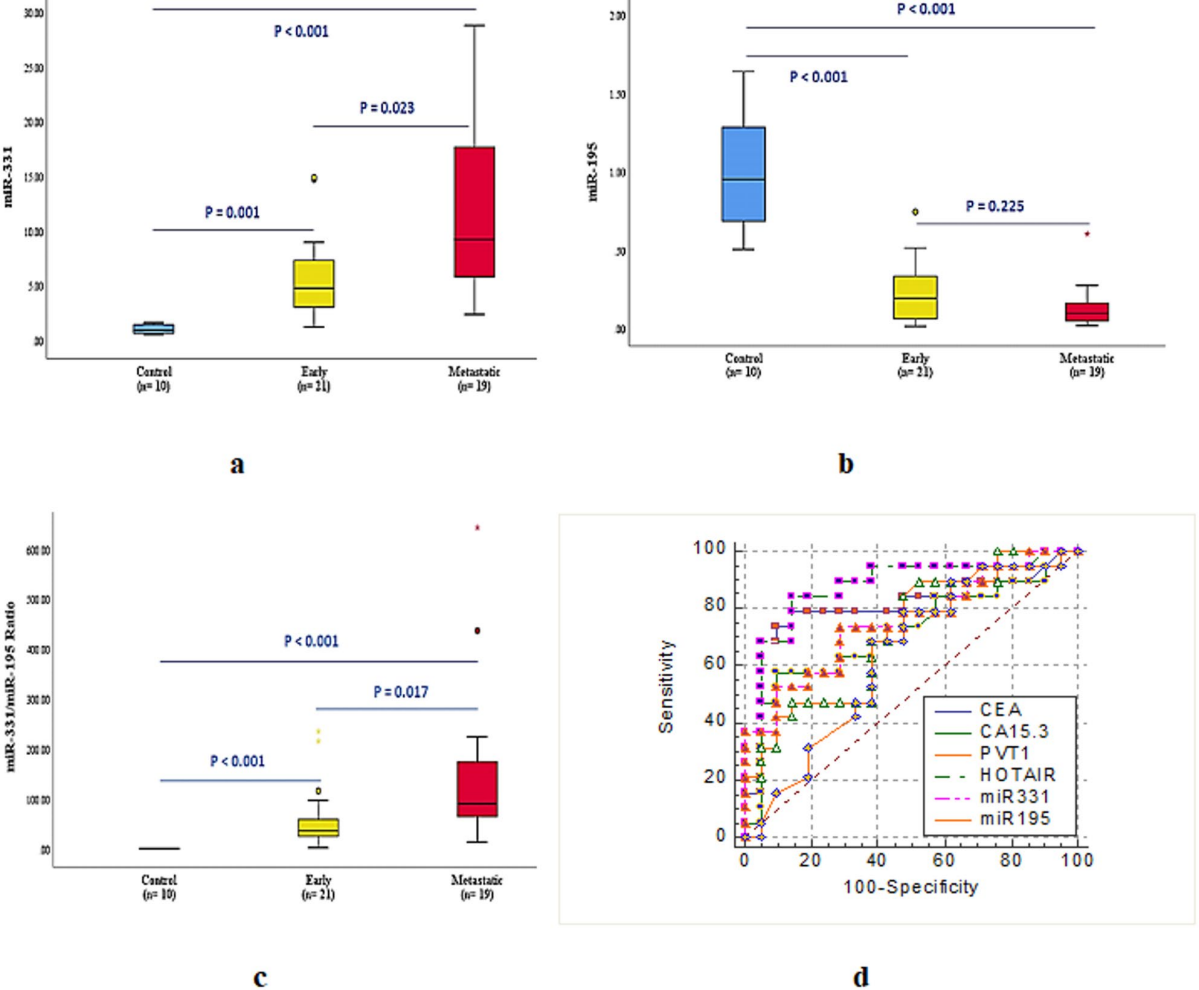
Differential circulating level of micro-RNAs as miR-331, miR-195 and miR-331/miR-195 ratio (**a**) miR-331 circulating levels; **b** miR-195 circulating levels and (**c**) miR-331/miR-195 ratio; and the efficacy of serum parameters as diagnostic biomarkers for early and metastatic breast cancer. ROC curve analysis illustrating the discriminatory power of CEA, CA 15.3, HOTAIR, PVT1, miR-331, miR-195 and miR-331/miR-195 in distinguishing between early and metastatic breast cancer

There are a marked significant higher serum miR-331 levels were detected in metastatic and early groups compared to control group, corresponding to an average fold-change 11.9 and 5.6 respectively. In addition to that, results showed also a significant higher miR-331 levels in the metastatic breast cancer patients compared to the early patients (Fig. 2a).

The circulatory miR-195 level significantly declined in the metastatic and early breast cancer patients compared to the control group with an average fold-change 0.13 and 0.22 respectively. While, there is no significant difference in miR-195 level between early and metastatic groups (Fig. 2b).

The calculated miR-331/miR-195 ratio showed marked significant increase in the two breast cancer groups compared to healthy controls, corresponding to an average fold-change 151.2 for metastatic patients and 58.1 for early breast cancer patients. In addition to that, this results showed significant increase of miR-331/miR-195 ratio in the metastatic breast patients compared to the early cancer groups (Fig. 2c).

Effect size analysis showed large discrimination effects for HOTAIR (AUC 0.881; *r* ≈ 0.62), moderate-to-large effects for miR-331 (AUC 0.754; *r* ≈ 0.48), and large effects for the miR-331/miR-195 ratio (AUC 0.774; *r* ≈ 0.52). PVT1 demonstrated small-to-moderate effects. These findings are preliminary due to limited sample size (Fig. 2d).

### Univariate and multivariate logistic regression analysis to predict Metastatic breast cancer (*n* = 21 vs. 19)

Univariate and multivariate logistic regression analyses were carried out to assess the predictive values of PVT1, HOTAIR, miR-331, miR-195, miR331/miR195 Ratio, CEA and CA15.3 in breast cancer.

In the univariate analysis, serum levels of PVT1, HOTAIR, MiR-331, miR331/miR195 Ratio, CEA and CA 15.3 were found to be significant predictors associated with risk of being metastatic breast cancer and after adjusted by Initial Stages [high III, IV] and Initial Regional Lymph Nodes (N3) using multivariate analysis, it was found they are independently can predict metastatic breast cancer (Tables 7 and 8).

**Table 7:** Univariate logistic regression analysis for the different markers and clinical data to predict metastatic breast cancer

	#Univariate	
	*p*	OR (LL – UL 95%C.I)
PVT1	0.028^*^	26.538 (1.427–493.49)
HOTAIR	0.003^*^	669528.1(98.73–4.5 × 10^9^)
miR331	0.010^*^	25.204 (2.173–292.299)
miR195	0.172	0.347 (0.076–1.586)
miR-331/miR-195 Ratio	0.010^*^	12.035 (1.816–79.780)
CEA	0.003^*^	7.648 (1.973–29.651)
CA15-3	0.015^*^	9.313 (1.555–55.763)
Age (Years)	0.640	1.016 (0.950–1.087)
Post menopause status	0.726	1.250 (0.358–4.363)
Histologic Type [IDC]	0.614	1.895 (0.158–22.751)
Initial Stages [high III, IV]	0.024^*^	5.000 (1.231–20.301)
Grade [III]	0.169	0.294 (0.051–1.683)
Lymph node status [Positive]	0.277	2.100 (0.551–8.002)
Lympho Vascular Invasion [Positive]	0.388	1.875 (0.450–7.821)
Initial Primary Tumor (T4)	0.894	1.125 (0.198–6.385)
Initial Regional Lymph Nodes (N3)	0.027^*^	6.909 (1.239–38.516)
Initial Regional Lymph Nodes [N1,2,3 (Positive)]	0.277	2.100 (0.551–8.002)
Initial Estrogen Receptor [Negative]	0.874	1.133 (0.241–5.340)
Initial Estrogen Receptor		
Negative		1.000
+	0.782	0.750 (0.098–5.768)
++/+++	0.922	0.923 (0.188–4.538)
Initial Progesterone Receptor [Positive]	0.835	1.172 (0.264–5.208)
Initial Progesterone Receptor		
Negative		1.000
+	0.819	1.250 (0.185–8.444)
++/+++	0.863	1.146 (0.244–5.391)
Initial HER2Neu Receptor [Negative]	0.585	1.500 (0.351–6.417)
Diagnosis [Left Breast Cancer]	0.517	1.512 (0.433–5.280)
Initial Tumor Size (˃2 cm)	0.388	1.875 (0.450–7.821)

*OR* Odd`s ratio, *C.I* Confidence interval, *LL* Lower limit, *UL* Upper Limit

#Logarithmic normalization

*Statistically significant at *p* ≤ 0.05

**Table 8:** Univariate and multivariate logistic regression analysis for the different markers adjusted by initial stages [high III, IV] and initial regional lymph nodes (N3) to predict metastatic breast cancer

	#Univariate		^#^Multivariate	
	*p*	OR (LL – UL 95%C.I)	*p*	OR (LL – UL 95%C.I)
PVT1	0.028^*^	26.538 (1.427–493.49)	0.040^*^	36.711 (1.178–1143.61)
HOTAIR	0.003^*^	669528.1(98.73–4.5 × 10^9^)	0.006^*^	1.026 × 10^6^ (53.684–2.0 × 10^10^)
miR331	0.010^*^	25.204 (2.173–292.299)	0.006^*^	76.848 (3.485–1694.768)
miR331-miR195 Ratio	0.010^*^	12.035 (1.816–79.780)	0.033^*^	12.326 (1.227–123.816)
CEA	0.003^*^	7.648 (1.973–29.651)	0.007^*^	8.025 (1.784–36.102)
CA15-3	0.015^*^	9.313 (1.555–55.763)	0.011^*^	21.615 (2.012–232.256)

*OR* Odd`s ratio, *C.I* Confidence interval, *LL* Lower limit, *UL* Upper Limit

#Logarithmic normalization

*Statistically significant at *p* ≤ 0.05

Progression-free survival and the prognostic potential of non-coding rnas in metastatic breast cancer: A Kaplan-Meier analysis in egyptian female patients

**Fig. 3:**
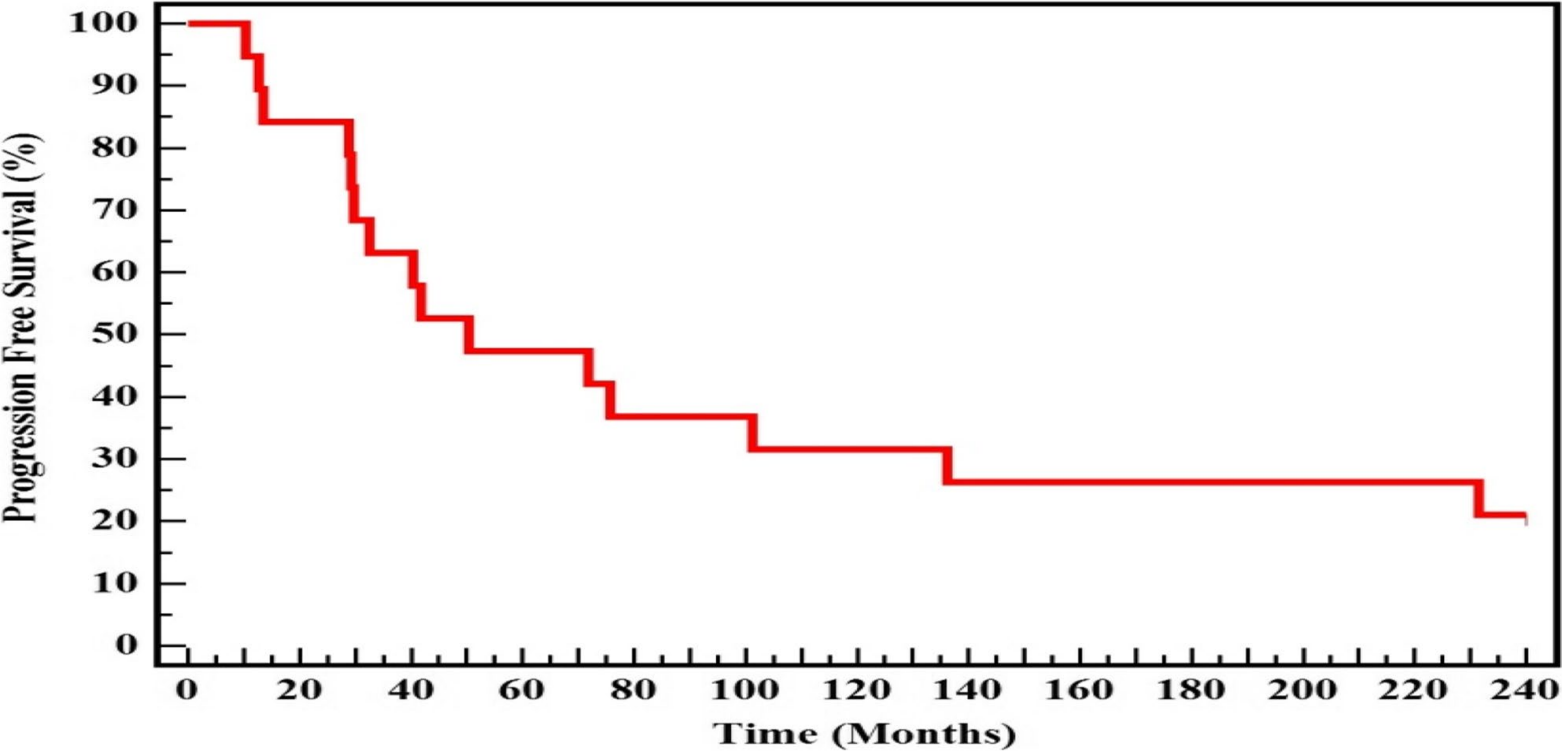
Kaplan-Meier survival curve for progression free survival (*n* = 19)

Kaplan-Meier analysis of progression-free survival (PFS) in a cohort of 19 metastatic breast cancer patients demonstrated strong short-term disease control, with 100% of patients remaining progression-free at 6 months and 94.7% at 1 year. However, PFS markedly declined to 21.1% by the end of the follow-up period, underscoring the aggressive and progressive nature of the disease. This pattern supports the potential utility of non-coding RNAs (ncRNAs) as prognostic biomarkers. Correlation of ncRNA expression with PFS may offer clinically relevant insights, particularly in Egyptian female patients, highlighting the importance of population-specific studies in advancing personalized cancer management (Table 9; Fig. 3).

**Table 9:** Kaplan-Meier survival curve for progression free survival (*n* = 19)

	Mean (months)	Median (months)	% 6 Month	% 1 year	% End Study
Progression free Survival	98.19	50.33	100.0%	94.7%	21.1%

Using observed effect sizes, future validation studies would require approximately 40–50 patients per group for HOTAIR and 55–65 per group for miR-331 or the miR-331/miR-195 ratio to achieve 80% power at α = 0.05. These calculations underscore the exploratory nature of this pilot study and support the need for larger cohorts.

## Discussion

This findings suggest that serum levels of PVT1, HOTAIR, miR-195, miR-331, and the miR-331/miR-195 ratio are promising biomarkers for breast cancer. These non-coding RNA markers warrant further investigation for their potential application in guiding therapy, predicting patient prognosis, and informing drug development strategies. Notably, HOTAIR, miR-331, and the miR-331/miR-195 ratio effectively distinguished metastatic breast cancer patients from those with early-stage disease. In contrast, conventional tumor markers such as CEA and CA 15.3 demonstrated a failure in providing prognostic utility in this study.

This results observed that serum CEA level was significantly increased in metastatic BC patients compared to both early and control group. But, there was no significant difference between early BC patients and control group. Zhang showed statistical differences in the concentration of CEA between the distant metastatic BC group and the non-metastatic BC group [32]. Also, Fu and Li investigated the associations between serum levels of CEA with BC susceptibility by a systematic meta-analysis and they found significantly elevated serum levels of CEA in patients with malignant BC, but not in those with benign tumors [33].

This results found that CEA serum level could significantly differentiate early from metastatic BC patients, with a sensitivety of 78.95%, a specificity of 85.71%, and an AUC of 0.807. This is in agreement with Mi et al. who found significant diagnostic value of CEA in distinguishing BC from healthy controls with an AUC of 0.810, sensitivity of 85.71% and specificity of 63.49% [34]. Other study demonstrated that CEA as a biomarker for BC had sensitivity 65.4%, specificity 50.0% with an AUC of 0.50 [35]. Also, Zhang et al. demonstrated that CEA had a diagnostic ability in discriminating distant metastasis from non-metastasis BC with 57.1% sensitivity, 87.4% specificity with an AUC of 0.755 [32].

CA15-3 is generally used as specific indicator of BC, which is of great significance for the diagnosis, prognosis, and the monitoring of curative effect and its significant increase is associated with bone metastasis. Zhang demonstrated the limitation of CA15-3 as a marker of BC was the lack of sensitivity to early patients, but the use of serum CA15-3 level with other markers could improve the sensitivity to the detection of recurrence and metastatic disease [32]. Other study demonstrated that, high serum CA15-3 levels are associated with higher BC stage, tumor size, positive axillary lymph nodes, and worse overall survival and disease-free survival [20]. This results revealed that serum CA15.3 level was significantly increased in metastatic BC patients compared to both early and control group. But, there was no significant difference between early BC patients and control group. In aggrement with study found significantly elevated serum levels of CA15-3 in patients with malignant BC, but not in those with benign tumors by using a systematic meta-analysis [33].

We also found that CA 15.3 could significantly discriminate early and metastatic BC patients, with a sensitivety of 57.89%, a specificity of 90.48%, and an AUC of 0.716. In agreement with [34] who found significant diagnostic value of CA15.3 in distinguishing BC from. And they also differentiate patients with early from advanced BC with 80% sensitivity, 86.7% specificity, 85.7% PPV, 81.3% NPV and 83.3% efficacy. Other study demonstrated that CA15.3 as a biomarker for BC had sensitivity 73.1%, specificity 60.0% with an AUC of 0.65 [35]. Zhang demonstrated that CA15-3 had a diagnostic ability in discriminating distant metastasis from non-metastasis BC with 59.2% sensitivity, 94.1% specificity with an AUC of 0.821. In addition, we found that CA15.3 serum level could be significant predictor associated with risk of being metastatic BC [32]. Other study [33], found that increased levels of CA15-3 may be one factor that predicts a poor prognosis. But, it may require combining multiple biomarkers to allow the detection of different subtypes in BC.

Many studies have shown that lncRNA PVT1 is related to the occurrence and development of many malignant tumors and it has promoted tumorigenesis and development in a many malignant tumors, such as gastrointestinal cancer, cholangiocarcinoma, gastric cancer, colorectal cancer, pancreatic cancer and BC [36]. PVT1 is abnormally overexpressed in many types of cancers which can interact with c-Myc and enhance tumor growth, progression, invasion, metastasis, and chemoradiotherapy resistance. The most common mechanism of PVT1 in many cancers is regulation of the relevant signaling pathways by competitive endogenous RNA (ceRNA), which promotes the presence and development of cancer [37]. Zhang et al. showed that the GG genotype of the single nucleotide polymorphism rs13281615 may affect the growth of BC via PVT1 and is associated positivity with estrogen receptor, higher tumor grades, and higher proliferative indices [38]. Another study demonstrated that PVT1 promoted cell proliferation in BC via the negative regulation of P21 [37]. Also, another study showed that PVT1 influenced Epithelial-mesenchymal transition (EMT) through P21, resulting in the proliferation and migration of triple-negative BC [37].

It was found that PVT1 may be a potential biomarker for BC prognosis [39]. The upregulation of sex determining region Y (SRY)-box 2 (SOX2) transcription factor which can bind to PVT1 promoter and enhanced its transcription lead to promotes BC progression through Epithelial-mesenchymal transition (EMT) [24]. PVT1 was shown to regulate cell proliferation and tumor growth in triple-negative BC through beta-catenin signaling pathway [40].

In the present study, the serum level of PVT1 was significantly increased in early and metastatic BC patients compared to control subjects with no significant difference between the metastatic and early group. Also, we also found that serum levels of PVT1 could significantly discriminate early and metastatic BC patients, with a sensitivety of 89.47%, a specificity of 47.62%, and an AUC of 0.718. This is in agreement with previous study which reported significant increase of PVT1 expression in BC patients compared to control group, with a sensitivety of 62%, a specificity of 64% and an AUC of 0.67 [22]. Also, Li et al. demostrated the diagnostic utility of PVT1 for BCs with the sensitivity and specificity at 51.2 and 75.0%, respectively and an AUC of 0.63 [41]. Liu et al. also showed that PVT1 levels were significantly increased in BC samples compared with either healthy controls or breast fibroma patients. In addition, they found no significant difference in BC patients compared to fibroadenoma [24].

This results in metastatic group revealed that PVT1 was significantly increased in patients with negative initial ER, and PR compared to patients with positive ER, and PR. Also, significant increase in TNBC compared to Luminal A, B. However, no significant association was found between PVT1 serum levels with other clinicopathological parameters in early group. El-Fattah et al. reported no significant asssociated between PVT1 expression and clinicopathological features in BC patients [22]. Also, other study showed no correlation between PVT1 level and clinicopathological feature in metastatic BC and non metastatic BC patients [42]. In this study, PVT1 level was found to be positively correlated with the level of other LN-RNA; HOTAIR in early and metastatic BC groups but previuos study of El-Fattah et al. observed no correlation between them [22]. In hepatocellular carcinoma, the PVT1 over expression was significantly correlated with vascular invasion, liver cirrhosis and TNM stage [43]. Other study showed high PVT1 expression exhibited greater lymph node metastasis, venous invasion compared with low PVT1 expression in colorectal cancer [44]. PVT1 also served as a vital regulator in the prognosis of various tumors, such as cervical carcinoma, ovarian cancer, and osteosarcoma [45]. In addition, PVT1 is upregulated in clinical TNB BC tumthiss [46]. This results demonestrated that there is a significant difference in PVT1 serum levels between early and metastatic groups in patients diagnosed with grade (X, II), patients with positive lymph nodes, patients with positive lympho vascular invasion and patients diagnosed at sampling with negative HER2 Neu Receptor. Other study demonstrated that PVT1 level was associated with cancer histological grade, and lymph node metastasis. Whereas, no significant correlation was found between the expression of PVT1 and other clinicopathological characteristics of patients, for example, expression of ER, PR and Her-2 in the tissue [41].

This results revealed that PVT1 serum level was found to be a significant predictor associated with risk of being metastatic breast cancer. This in agreement with Liu et al. who suggested that PVT1 was a recommended predictor for BC diagnosis and staging assessment [24]. Because the PVT1 expression was the highest in cell line Hs578t and MCF-7 in his study [41]. also demonstrated that multivariate Cox proportional hazards regression analysis was performed to identify independent predictors of survival, his results showed that PVT1 expression was significantly correlated with disease-free survival and overall survival.

The HOTAIR is the first lncRNA identified in correlation with a poor prognosis in BC. It has a wide-range impact on BC development, metastasis, and treatment resistance [47]. HOTAIR is cell cycle-associated gene as it promotes the cell cycle passing through the restriction point during the G1 phase by regulating CDK4/6-cyclin D and the Rb-E2F pathway [48]. The expression of HOTAIR in epithelial cancer cells induced alteration of histone H3 lysine 27 methylation, gene expression, and increased cancer invasiveness and metastasis [49]. In BC, HOTAIR and BC gene 1 (BRCA1) are able to bind the subunit of EZH2 (Enhancer of Zeste 2 Polycomb Repressive Complex 2 Subunit) coordinating the PRC-dependent epigenetic regulation of the chromosome. The promoter of the HOTAIR gene can also be bound by interferon regulatory factor-1 (IRF1) and inhibit its expression in BC cells. It is known that HOTAIR is also associated with an aberrant DNA methylation profile in cancer [48]. Other study demonstrated that HOTAIR regulates HER2 expression by sponging miR-331-3p in gastric cancer [50].

In this study, the serum level of HOTAIR was significantly elevated in early and metastatic BC patients compared to healthy control subjects. In addition, we found significant increase of HOTAIR levels in metastatic group compared to early group and its level could significantly discriminate early and metastatic BC patients, with a sensitivety of 84.21%, a specificity of 85.71%, and an AUC of 0.881. This is agreement with previous study which reported significant increase of HOTAIR expression in BC patients compared to control group and showed significant increase in BC patients compared to fibroadenoma. The study also showed that HOTAIR level significantly differentiate between BC patients and control subjects, with a sensitivety of 62%, a specificity of 64% and an AUC of 0.65. In addition its level significantly differentiate between BC patients and fibroadenoma patients, with a sensitivety of 76%, a specificity of 76% and an AUC of 0.77 [22]. Also, another study showed significant increase in the expression levels HOTAIR in the plasma of BC patients compared to healthy female controls [51]. The study of Zhang et al. found that the diagnostic value of plasma HOTAIR as a potential biomarker for BC with sensitivity 69.2%, specificity 93.3% and an AUC of 0.80, which indicated that HOTAIR had the strongest ability to distinguish BC from healthy individuals [35]. Recently reported that, serum HOTAIR levels demonstrated a good ability to discriminate tumors larger than 5 cm from smaller tumors, with 86% sensitivity and 68% specificity (AUC = 0.797), in addition, HOTAIR levels distinguished patients with stage III/IV BC from those with earlier stages, exhibiting 75% sensitivity and 64% specificity (AUC = 0.698) [52].

In this results, HOTAIR serum level in early BC group had significant difference for comparing initial primary tumor T0,T1,T2 to initial primary tumor T3,T4. This is agreement with previous study which reported significant association between HOTAIR high expression and advanced T stages in head and neck squamous cell carcinoma [53]. We also found that, HOTAIR level in metastatic BC group had significant higher serum levels in TNBC than luminal A and B. But, Abdel-Hamid et al. revealed that higher HOTAIR levels found in tumors of the luminal B molecular subtype compared to other molecular subtypes and in patients with estrogen receptor positivity (ER+), although this difference did not reach statistical significance [52]. This results showed significant higher level of HOTAIR in metastatic BC group than in early BC group of the following subgroups: Grade (X, II), positive or negative lymph nodes, positive lympho vascular invasion and negative HER2Neu at sampling.

Other study assess the association between HOTAIR expression levels and lymph node metastasis, they showed that the patients with high levels had a higher incidence lymph node metastasis compared with that in patients with low levels [54]. In addition, Collina et al. analyzed the expression of HOTAIR in TNBC cases and found no significant association with clinical pathological parameters, such as grade and stage but found a strong association with lymph node metastases [55]. Other study demonstrated that HOTAIR expression significantly correlated with lymph node metastasis, estrogen receptor, and triple positive in plasma BC patients [35]. Conversely, El-Helkan et al. showed no correlation between HOTAIR expression with clinicopathological feature in metastatic BC and non metastatic BC patients [42].

This results also revealed that HOTAIR serum level was found to be a significant predictor associated with risk of being metastatic BC. In agreement with Cantile et al. who reported the aberrant expression of HOTAIR in primary BC tumors with high metastatic potential and poor survival, suggesting HOTAIR as a powerful predictor of BC tumor progression [48]. In addition, Tang et al. showed that serum exosomal HOTAIR levels have the potential to be a prognostic and diagnostic biomarker for BC patients [56].

In the present study, the serum level of miR-331 was significantly higher in BC patients compared with the control group, and significantly higher in metastatic BC group compared with the early BC group and its levels could significantly discriminate early and metastatic BC patients, with a sensitivety of 73.68%, a specificity of 71.43%, and an AUC of 0.754. This in agreement with the study of Papadopoulos et al. which demonstrated that miR-331 was significantly upregulated in malignant specimens in comparison to their benign counterparts. Also, this study showed significant increase in miR-331 level in early BC group compared to healthy control group. Also, miR-331 levels could discriminate between breast tissue specimens of malignant and benign tumors by providing an AUC of 0.597 [57]. This is agreement with McAnena et al. who reported significant increase of miR-331 expression in metastatic group compared to both locally confined BC group and healthy control group and demonstrated that the AUC of 0.902 was achieved combining miR-331 and miR-195 with sensitivity of 95% and specificity of 76% [58].

This data revealed that serum miR-331 level could related to the clinicopathological status of the patitents as it was significantly higher in patient with negative lymph nodes than positive lymph nodes in early BC group. Also we found significant relation between miR-331 level and patients with Grade (X, II) and patients with Positive lympho vascular invasion between early and metastatic BC groups. But, McAnena et al. demonstrated that no significant relation between miR-331 level and clinicopathological status in locally confined BC group between positive and negative lymph nodes [58]. We found significant relation between miR-331 level and patients with positive lymph nodes and patients who had initial regional lymph nodes (N1) between early and metastatic BC groups. Similarly, Yang et al. demonstrated that abnormal miR-331 expression was correlated with lymph nodes metastasis and TNM stage in gastric cancer patients [59]. This results aslo showed significant relation between miR-331 level and patients diagnosed at sampling with negative HER-2Neu receptor between early and metastatic BC groups. Shee et al. demonstrated that MiR-331-3p (which is a member of the miRNA-331 family) has been found to down-regulate HER-2 in BC cells. Also, miRNA-331-3p was found to be involved in adenocarcinoma of prostate and glioblastoma by regulating the expressions of epidermal growth factor receptor and HER-2 via reducing Akt activity [60]. In addition, we found that miR-331 serum level could be significant predictor associated with risk of being metastatic breast cancer. This in agreement of previous study which demonsrated that expression of miR-331-3p in tumor tissues could be an independent prognostic marker for non-small-cell lung cancer patients [61].

Previously, miR-195 has been used as a diagnostic biomarker of BC, it was investigated as a tumthis suppressor. Several studies found that, miR-195 has been shown to target Bcl-2, inducing apoptosis, and target FASN, HMGCR, ACACA and CYP27B1 in hormone-receptor positive BC cell lines, suppressing tumthis growth, EMT, invasion and metastasis [62, 63]. Other studies have shown that mir-195 regulates biological processes such as cell proliferation and cell cycle by targeting CDK4, CDK6, cyclin D1 and others [58, 64–66]. reported that miR-195 inhibited cell cycle transition of G1 to S phase by directly targeting cyclin E1, where E2F plays a critical role. E2F interacts with other factors such as cyclin D, cyclin E, and cyclin-dependent kinases, to suppress the G1 to S phase transition of the cell cycle. Previous study [67], reported that miR-195 expression was negatively correlated with the degree of malignancy of pathological tissues of the tumor in the Cardiac Carcinoma patients. Other study found that PVT1 could decrease miR-195 expression via enhancing histone H3K27me3 in the miR-195 promoter region and also via direct sponging of miR-195 in cervical cancer cells [68].

Another important miRs that was assayed in this study is miR-195 which was significantly lower in metastatic BC group compared to healthy control group. But, there was no significant difference in miR-195 level between early and metastatic BC groups, however, miR-195 could significantly discriminate early and metastatic BC patients, with a sensitivety of 78.95%, a specificity of 52.38%, and an AUC of 0.635. Previous study showed significant decrease of miR-195 level in metastatic group compared to both locally confined BC and healthy control groups [58]. Other study found that miR-195 expression was downregulated in BC compared with normal breast tissue, which is consistent with the role of miR-195 as a tumor suppressor miR [66]. Also, a significant decline in miR-195 expression levels was reported in BC patients in comparison to healthy controls [69]. Cecene at al analyzed miR-195 expression in Luminal A and B BC tissues and found downregulation of its expression in BC with respect to normal adjacent tissues [70]. In addition, we observed that miR-195 level was significantly lower in early BC group compared to control group. Fan et al. reported AUC values for circulating miR-195 in distinguishing patients with BC from healthy controls was 0.964 with sensitivity of 100% and specificity of 80% [71]. Also, it was found that miR-195, at a cut off value of 0.996, sensitivity was 90%, specificity was 100% with an AUC of 0.987 [72]. Another study reported ROC curve analysis of miR-195 (AUC of 0.672) showed better diagnostic accuracy and miR-195 showed high sensitivity values 77.8% [69]. Hamam et al. showed that miR-195 could differentiate BC cases from controls, with sensitivity of 87.7%, specificity of 91%, with an AUC of 0.937 [73]. However, McAnena et al. showed no significant difference in miR-195 expression between the locally confined BC and healthy control group [58].

We found in metastatic group significant elevation of miR-195 levels in patients with stages I, II compared to stages III, IV. previous study demonstrated that the serum levels of circulating miR-195 was significantly increased in patients with early-stage BC (stage I or II) in comparison with healthy controls [71].

In addition, this results found significantly lower miR-195 levels in BC patients with positive lymph nodes compared to negative lymph nodes This is in agreement with Wang et al. who found that the miR-195 expression in pathological tissues of cardiac carcinoma in patients with lymph node metastasis was significantly lower than that in patients without lymph node metastasis and the number of lymph node metastases was negatively correlated with the expression level of miR-195 [67].

In early BC group, this results showed significantly lower miR-195 levels in initial primary tumor T3,T4 compaired to T0,T1,T2. In addition, mir-195 was significant increased in early BC group than metastatic BC group in those who presented initially with regional lymph nodes (N1) But, This results showed no relation between miR-195 level and patients with Grade (X, II), Positive/negative lymph nodes status, positive lympho vascular invasion and patients diagnosed at sampling with negative HER-2Neu receptor between early and metastatic BC groups. In agreement with McAnena et al. who demonstrated that no significant difference for miR-195 level in locally confined BC group between positive and negative lymph nodes, between tumthis grades (I-III) and between lymphovascular invasion status (positive/negative) [58]. Previous study found low serum miR-195 level related to distant metastasis, lymph node metastasis, histological grade and TNM stage. Also, the study revealed that higher CA15-3 and lower miRNA195 were considered as independent predictors for BC susceptibility. Also they found that miR-195 was considered as an independent predictor for higher BC tumthis grade and advanced stage [72]. Other studies reported that, miR-195 associated with advanced stage, higher tumor grade, negative hormone receptors, and the Triple-negative subtype in BC patients from different populations, including Indian and Saudi Arabian females [74].

In this study we calculated a new index; miR-331/miR-195 ratio, to combine the increased levels of miR-331 and the declining levels of miR-195 in order to improve the sensitivity and specificity of miRNA assay for use as diagnostic biomarkers. The ratio-based miRNA models may be especially effective because their design eliminates the need for additional normalisation variables [75]. Pervious study demonstrated that the importance of miRNA ratios in distinguishing early stage BC from benign lesions. They chose the miRNA ratio over the single miRNA to provided more candidates for diagnosis of early stage BC [76]. This results showed marked elevation of the miR-331/miR-195 ratio in BC patients compared to healthy control group and the metastatic group have significantly higher ratio compared to early group. In addition, the ratio showed high AUC value of 0.774 in the present study, and would be satisfactory for clinical application. The ratio of miR-331/miR-195 in serum could distinguish early BC patients from metastatic BC patients with 78.95% sensitivity and 76.19% specificity which are better values than any of the two miRs alone.

Between early and metastatic groups, we found a strong relation between miR-331/miR-195 ratio and patients with grade (X, II), patients with positive lymph nodes, patients with positive lympho vascular invasion and patients diagnosed at sampling with negative Her2-Neu receptor. These relationsa are missed with the single miR-195 in this study. Also, the results revealed that miR-331/miR-195 ratio is a sinificant predictor associated with risk of being metastatic breast cancer. Similarly, Fang et al. found that combination of miRNA ratios had a high diagnostic value for BC prediction [76].

### Limitation

The main limitation of this study is the relatively small sample size, which may restrict the generalizability of the findings. Although the current cohort provided meaningful preliminary evidence on the differential expression of circulating non-coding RNAs in early and metastatic breast cancer, larger and more diverse patient populations are required to validate these results. Future studies with expanded sample sizes and multi-center recruitment are recommended to confirm the diagnostic and prognostic potential of the investigated biomarkers.

## Conclusion

This pilot study provides preliminary evidence that circulating PVT1, HOTAIR, miR-331, and miR-195 may serve as potential diagnostic and prognostic biomarkers for breast cancer. Among these, HOTAIR, miR-331, and the miR-331/miR-195 ratio demonstrated significant differential expression between early-stage and metastatic patients, with HOTAIR showing the strongest discriminatory potential in ROC analysis. Additionally, the combination of PVT1, HOTAIR, miR-331, and the miR-331/miR-195 ratio appeared to differentiate metastatic from early-stage breast cancer in patients with higher-grade tumors, positive lymph nodes, positive lymphovascular invasion, and negative HER2 status. However, these findings are preliminary and hypothesis-generating, and further validation in larger, independent, and prospectively-collected cohorts is required before any clinical relevance can be inferred.

## Data Availability

All data produced in this study can be made available from the corresponding author upon a reasonable request.
